# Early pregnancy peripheral blood gene expression and risk of preterm delivery: a nested case control study

**DOI:** 10.1186/1471-2393-9-56

**Published:** 2009-12-10

**Authors:** Daniel A Enquobahrie, Michelle A Williams, Chunfang Qiu, Seid Y Muhie, Kimberly Slentz-Kesler, Zhaoping Ge, Tanya Sorenson

**Affiliations:** 1 Department of Epidemiology, University of Washington, Seattle, WA, USA; 2 Swedish Medical Center, Seattle, WA, USA; 3 Walter Reed Army Institute of Research, Silver Spring, MD, USA; 4 Almac Diagnostics, Durham, NC, USA

## Abstract

**Background:**

Preterm delivery (PTD) is a significant public health problem associated with greater risk of mortality and morbidity in infants and mothers. Pathophysiologic processes that may lead to PTD start early in pregnancy. We investigated early pregnancy peripheral blood global gene expression and PTD risk.

**Methods:**

As part of a prospective study, ribonucleic acid was extracted from blood samples (collected at 16 weeks gestational age) from 14 women who had PTD (cases) and 16 women who delivered at term (controls). Gene expressions were measured using the GeneChip^® ^Human Genome U133 Plus 2.0 Array. Student's T-test and fold change analysis were used to identify differentially expressed genes. We used hierarchical clustering and principle components analysis to characterize signature gene expression patterns among cases and controls. Pathway and promoter sequence analyses were used to investigate functions and functional relationships as well as regulatory regions of differentially expressed genes.

**Results:**

A total of 209 genes, including potential candidate genes (e.g. PTGDS, prostaglandin D2 synthase 21 kDa), were differentially expressed. A set of these genes achieved accurate pre-diagnostic separation of cases and controls. These genes participate in functions related to immune system and inflammation, organ development, metabolism (lipid, carbohydrate and amino acid) and cell signaling. Binding sites of putative transcription factors such as EGR1 (early growth response 1), TFAP2A (transcription factor AP2A), Sp1 (specificity protein 1) and Sp3 (specificity protein 3) were over represented in promoter regions of differentially expressed genes. Real-time PCR confirmed microarray expression measurements of selected genes.

**Conclusions:**

PTD is associated with maternal early pregnancy peripheral blood gene expression changes. Maternal early pregnancy peripheral blood gene expression patterns may be useful for better understanding of PTD pathophysiology and PTD risk prediction.

## Background

Preterm delivery (PTD) is a significant global public health problem affecting ~12-15% of all deliveries [1]. It is associated with greater risk of mortality and morbidity in infants and mothers and significant financial burden [2].

PTD is a complex cluster of problems associated with socio-economic, socio-demographic, socio-behavioral, environmental, medical, biological and genetic risk factors [3-8]. A number of pathophysiological pathways including chronic systemic inflammation and infection, endothelial dysfunction, oxidative stress, placental ischemia and reperfusion and neuro-endocrine alterations have been identified to be associated with PTD [6,9,10].

Variations in a number of candidate genes (e.g. TNF-alpha, IL-1RA, IL4R, MMP9, MMP1, TLR4) have been identified in both human and experimental studies [1,2]. Due to the diverse pathopysiologic processes associated with PTD, single candidate gene profiles may not adequately portray the multitude of pathogenetic changes associated with PTD. In addition, substantial evidence has been described for contributions of gene-environment interactions to the incidence of PTD [11,12]. Since gene expressions are influenced by both genetic variations and environmental exposures, global gene expression profiling studies may help to generate testable hypothesis related to the role of genes across the genome and potential gene-environment interactions in PTD.

Previous studies primarily evaluated gene expression on tissue, principally placenta collected after delivery [13-18]. Since evaluation of early pregnancy tissue changes on placenta in relation to PTD is associated with risk, most studies on placenta before delivery were experimental. Thus, inference on temporal relationships was limited. Peripheral blood gene expression profiling, which can be done at any point in pregnancy, is associated with minimal risk and has shown promising results in investigations of other disease processes (such as cancer, cardiovascular diseases, and environmental health) [19-23]; and, could be applied to investigations of PTD.

Substantial evidence, including work from our group, supports the thesis that pathophysiologic changes, including system pathologies, leading up to PTD start early in pregnancy [24-26]. Evaluating early pregnancy peripheral blood genome-wide expression will help add to the prognostic value of traditional parameters, identify potential biological targets for prevention and treatment, improve discrimination of sub-groups in a complex pathology such as PTD and develop diagnostic tests.

In this pilot case control study, nested within an ongoing prospective study, we investigated early pregnancy peripheral blood genome-wide gene-expression profiles of 14 women who had PTD and 16 women who had term delivery. We evaluated transcriptional gene expression patterns associated with PTD in an effort to develop predictive tools for PTD. Functions and functional relationships of differentially expressed genes were investigated to better understand pathophysiologic processes underlying PTD. Finally, promoter sequences analysis was conducted to investigate whether common transcription factors could account for co-expression of genes associated with PTD.

## Methods

### Overview

In a pilot nested case control study among participants of an ongoing prospective cohort study, we investigated early pregnancy differential peripheral blood gene expression among women who delivered preterm and controls who delivered at term.

### Study population

The study was conducted using information collected from participants of an ongoing prospective cohort study, the Omega study, conducted at the Center for Perinatal Studies (CPS) at Swedish Medical Center in Seattle, Washington. The Omega study was designed to examine metabolic and dietary predicators and other risk factors of pregnancy complications including preeclampsia, gestational diabetes mellitus and preterm labor. Selection of study participants has been described before [27,28]. Briefly, participants were recruited from women attending prenatal care clinics affiliated with Swedish Medical Center. Women who initiate prenatal care prior to 20 weeks gestation were eligible to participate. Ineligibility criteria included younger than 18 years of age, not speaking or reading English, not planning to carry the pregnancy to term, and/or not planning to deliver at the Swedish Medical Center. More than 80% of approached women consented to participate and more than 95% were followed through pregnancy completion. PTD was defined as delivery before completion of 37 weeks of gestation after pregnancy lasting at least 20 weeks of gestation (PREBIC Genetics Working Group) [3]. Estimated date of conception (EDC) assessed using maternal report of last menstrual period (LMP) combined with ultrasound at ≤20 weeks gestation was used to determine PTD. Women with multi-fetal gestation and women delivering infants with malformations were excluded. Controls were selected among women who completed 37 weeks of gestation frequency matched for maternal age and gestational age at blood collection. We initially identified 16 cases and 16 controls for the study. The procedures used in the Omega Study are in agreement with the protocols approved by the Institutional Review Board of Swedish Medical Center. All participants provide written informed consent.

### Data Collection

Information on risk factors was collected using in-person interviews, blood collection and medical records abstraction. Following enrollment, in-person interviews were conducted to collect data on socio-demographic characteristics, occupation, reproductive and medical histories, alcohol and tobacco consumption, environmental tobacco smoke exposure, medications, height, weight, physical activity, and familial histories of medical conditions. At or near the time of in-person interviews (16 weeks of gestation on average), trained phlebotomists collected 2.5 mLs (in duplicates) of antepartum peripheral blood from each participant in PAXgene™ Blood RNA tubes, part of a PAXgene™ Blood RNA System (PAXgene Blood RNA Kit) (PreAnalytiX, Qiagen, Inc) [29]. The PAXgene™ Blood RNA System enables consolidation of key steps of peripheral blood collection, nucleic acid stabilization and RNA purification to reduce RNA degradation, inhibit or eliminate gene induction and make possible long-term storage of peripheral blood samples. Further, the system allows for multiple Blood RNA tube freeze-thaw cycles without affecting RNA yields. Samples were immediately aliquoted and stored at -80°C until RNA extraction and analysis. After delivery, trained personnel abstracted data from Omega participants' maternal and infant medical records to ascertain pregnancy outcomes.

### Total RNA extraction

The PAXgene Blood RNA Kit (PreAnalytiX, Qiagen, Inc) was used for extraction and purification of total RNA from peripheral blood, according to manufacturers' protocols. The procedure, briefly, was as follows. Initial equilibration of samples to room temperature for two hours was followed by centrifugation of cell lysates. The pellets were washed and resuspended in optimized buffers and incubated with proteinase K to bring about protein digestion. Centrifugations through the PAXgene Shredder spin columns were carried out to homogenize the cell lysate and remove residual cell debris. The supernatant of the flow-through fraction was then transferred to a fresh microcentrifuge tube. Ethanol was added to adjust binding conditions and the lysate was applied to a PAXgene RNA spin column. During centrifugation, RNA was selectively bound to the PAXgene silica membrane as contaminants pass through. Remaining contaminants were removed in several efficient wash steps. Between wash steps, the silica-membrane is treated with DNase I to remove any residual DNA contaminants. After wash steps, pure RNA samples were eluted in a buffer, heat denatured at 65°C and immediately chilled on ice. Extracted RNA was stored at -80°.

### RNA quantity measurement and quality control

Total RNA concentration was determined by ultraviolet absorbance at 260 nm (A_260_) by direct measurement (i.e non-diluted) on a NanoDrop ND1000 spectrophotometer (ThermoFisher Scientific, Wilmington, DE). Purity of RNA was assessed by evaluating readings at 260 nm and 280 nm (A_260_/A_260_) that provides an estimate of purity of RNA with respect to contaminants that absorb UV, such as protein. All samples had A260/280 values greater than 2.0 indicating a high level of purity. Agilent 2100 Bioanalyzer-based quality control criteria were also used to measure RNA quality (Agilent Technologies Inc, Palo Alto, CA). The RNA Integrity Number (RIN), based on a 10 point scale, is an objective measure of total RNA quality and integrity based on several features of the RNA profile (including the fraction of the sample in the region of 18S and 28S RNA and the relative height of the 28S peak). One sample which had met the spectrophotometric QC criteria (A260/A280 = 2.04), had poor results on the bioanalyzer (RIN = 2.3) indicating a high level of RNA degradation and was therefore deemed to be of insufficient quality for gene expression measurement. Samples were then kept in frozen storage at -80°C. All RNA samples, including reference RNAs, underwent quality control checks and were labeled using the same standardized protocols.

### RNA amplification, fragmentation and labeling

All sample target preparations were conducted in accordance with the guidelines detailed in the technical manuals for the NuGEN™ Ovation™ Whole Blood Reagent and NuGEN™ Ovation™ RNA Amplification System V2 (amplification) in combination with the NuGEN™ FL-Ovation™ cDNA Biotin Module V2 (fragmentation and labeling). Briefly, these steps are described below.

Total RNA (50 ng) was amplified using the NuGEN™ Ovation™ Whole Blood Reagent and NuGEN™ Ovation™ RNA Amplification System V2. First-strand synthesis of cDNA was carried out using a unique first-strand DNA/RNA chimeric primer mix, resulting in cDNA/mRNA hybrid molecules. Following fragmentation of the mRNA component of the cDNA/mRNA molecules, second-strand synthesis was carried out and double-stranded cDNA was formed with a unique DNA/RNA heteroduplex at one end. In the final amplification step, RNA within the heteroduplex was degraded using RNaseH, and replication of the resultant single-stranded cDNA was achieved through DNA/RNA chimeric primer binding and DNA polymerase enzymatic activity. The amplified single-stranded cDNA was purified using the Zymo Research Clean and Concentrator™-25 Kit to allow for accurate quantitation of the cDNA and to ensure optimal performance during the fragmentation and labeling processes. The single stranded cDNA was assessed for quality using spectrophotometric methods in combination with the Agilent Bioanalyzer. For the labeling step, 4.4 μg of amplified single-stranded cDNA was fragmented and labeled using the FL-Ovation™ cDNA Biotin Module V2. The enzymatically and chemically fragmented product (50-100 nt) was labeled via the attachment of biotinylated nucleotides onto the 3'-end of the fragmented cDNA.

### Hybridization, Washing and Staining

The resultant fragmented and labeled cDNA was added to the hybridization cocktail in accordance with the NuGEN and Affymetrix guidelines for hybridization onto Affymetrix Human Genome U133 Plus 2.0 GeneChip^® ^Arrays (Affymetrix, Sunnyvale, CA). This array platform offered a comprehensive coverage (comprised of more than 54,000 probe sets and 1,300,000 distinct oligonucelotide features representing over 47,000 transcripts and variants, including 38,500 well-characterized human genes) of the transcribed human genome on a single array. Following hybridization for 18 hours at 45°C, the arrays were washed and stained on the GeneChip^® ^Fluidics Station 450 (Affymetrix, Sunnyvale, CA) using the appropriate fluidics script, before being inserted into the Affymetrix autoloader carousel and scanned using the GeneChip^® ^Scanner 3000 (Affymetrix, Sunnyvale, CA). All scanned array images were visually inspected for chip surface artifacts that could adversely impact the data, and then data from each array was quantified using GeneChip^® ^Operating Software (Affymetrix, Sunnyvale, CA). GCOS automatically acquires and analyzes image data, defines probe cells and computes an intensity value for each probe cell. These intensity values were pre-processed for analysis using quality control filters and normalization.

### GeneChip quality controls and normalization

A number of quality control checks were conducted to evaluate GeneChip quality. First, background values (which can range from 20 to 100) of GeneArray scanners calibrated to the new PMT setting (10% of maximum) were assessed for comparability. Second, the GAPDH gene was used to assess RNA sample and assay quality specifically for the GeneChip array. The signal values for the 3' probe set for GAPDH were compared to the 5' probe set. The ratio of the 3' to the 5'probe sets were expected to be no more than 3. Third, hybridization controls on the GeneChip array (four *E. Coli *genes, bioB, bioC and bioD and the cre gene) were spiked into each sample independent of RNA sample preparation to evaluate hybridization efficiency. Fourth, raw noise (Q value), a measure of pixel-to-pixel variation of probe cells on a GeneChip array due to operation-associated electrical noise of the GeneArray scanner (unique to each scanner) was evaluated. Arrays that were scanned on the same scanner should ideally have comparable Raw Q values. Fifth, PolyA control genes, comprised of the dap, lys, phe, thr and trp genes from B. subtilis, were amplified and spiked into the RNA samples prior to amplification to serve as internal control genes. Finally, data were normalized using an error-weighted model with Rosetta Resolver Error Models (Rosetta, Seattle, WA) [30]. In order to distinguish 'signal' and 'noise' intensity, background filtering was done by removing error model based "absent-called" sequences and those with intensity values lower than 2× standard deviations of chip background.

### Real time quantitative polymerase chain reaction (RT-qPCR) experiment

A real time quantitative polymerase chain reaction (RT-qPCR) experiment was conducted to measure expression of nine selected genes that were differentially expressed in peripheral blood collected from women who delivered preterm as a confirmatory step of microarray based gene expression measures. In the first step, 1 μg total RNA from each of the 30 samples was reverse transcribed using the Transcriptor first strand cDNA synthesis kit (Roche Applied Science, Indianapolis, IN) according to manufacturer's instructions. Briefly, 1 μl of anchored-oligo (dT) 18 primer (2.5 μM final concentration) was added to each sample and heated at 65°C for 10 min and immediately cooled to 4°C. A mastermix was prepared on ice for all the samples with final concentrations of 1× Transcriptor reverse transcriptase reaction buffer (containing 8 mM MgCl2), 20 U of Protector RNase inhibitor, 1 mM each dNTP and 10 U of Transcriptor reverse transcriptase. The samples were mixed by pipetting and incubated in a thermocycler at 55°C for 30 min followed by 85°C for 5 min to inactivate the reverse transcriptase. Additional 4 samples were selected from the 30 in which the Transcriptor reverse transcriptase was replaced by PCR grade water and used to perform reverse transcription negative control reactions. The concentration of each reverse transcription was determined spectrophotometrically and diluted to 40 ng/μl using PCR-grade water for use as template for qPCR. The qPCR reactions were performed using the Roche LightCycler 480^® ^Probes kit and the LightCycler 480^® ^instrument according to the Lightcycler 480^® ^probes master protocol. Pre-designed exon spanning Taqman^® ^assays for each gene target were obtained from Applied Biosystems (Foster City, CA).

Each individual assay was run on an individual 96-well plate in duplicate for each sample; and, 2 reverse transcription negative controls and 2 no template control wells were included with each assay. A mastermix was prepared for each assay on ice to enable a final reaction concentration of 1× primer-probe mix and 1 Lightcycler 480^® ^Probes Master. Twelve μl of mastermix was dispensed into the appropriate wells of a Lightcycler 480^® ^96 well plate and 8 μl of template cDNA at 40 ng/μl was added. The plate was sealed and centrifuged for 2 min at 1,500 × g. The Lightcycler PCR was set-up for single FAM labeled probes and underwent an initial pre-incubation at 95°C for 10 min followed by 45 cycles of 95°C for 10 sec and annealing and extension at 60°C for 30 sec with fluorescence captured following each extension step. Assay sensitivity, reproducibility and transcript concordance with microarray platforms and primer efficiency were assessed and assay optimization was carried out to achieve better sensitivity, specificity, reproducibility and a wider linear dynamic range.

Individual reactions were characterized by the PCR cycle at which fluorescence first rises above threshold background fluorescence (the threshold cycle, Ct). The assay relies on fluorescence signal, which is proportional to the amount of DNA produced during each PCR cycle. This correlation between fluorescence and amount of amplified DNA permits accurate quantification of target over a wide dynamic range, while retaining high sensitivity and specificity making this experiment useful as a confirmatory tool for microarray gene expression measurements. Samples were processed simultaneously to avoid batch-to-batch variation. Normalization of expression measurements were done using three reference genes (RHOA, KHDRBS1 and BAT1) which were selected based on their non-variant gene expression in the microarray platform across cases and controls.

### Data analysis

After data normalization, principle components analysis (PCA) was used to identify significant outliers based on expression measurements. Based on this analysis, one sample (PTD case), was identified as a significant outlier was removed from further analysis. Thus, for the final analytic set included 14 PTD cases and 16 controls.

Fold change of comparison for each sequence across the two groups (cases and controls) based on normalized data together with Student's t-test (two sample, unequal variances) were performed in order to identify differentially expressed genes. Volcano plot was used to depict distributions of fold change (log2 [fold change]) for biological significance and Student's t-test p-values (-log10 [p-value]) for statistical significance. Genes that met cutoffs of absolute fold change differences ≥ 1.5 and Student's t-test (p-values < 0.05) constituted the set of differentially expressed genes associated with PTD. Two-Dimensional hierarchical clustering analysis (2-D), using Cluster and TreeView softwares that employ a hierarchical clustering approach based on Pearson's correlation coefficient and PCA techniques were used to evaluate whether differentially expressed genes cluster arrays into groups (PTD and term delivery groups) [31,32].

Relationships between differentially expressed genes were investigated in pathway analysis using Ingenuity Pathway Analysis (IPA) software (Ingenuity, Redwood City, CA). In IPA, Ingenuity Pathways Knowledge Base (IPKB), a published and peer-reviewed database and computational algorithms will be used to identify local networks that are particularly enriched for the Network Eligible Genes, defined as genes in our list of differentially expressed genes with at least one previously defined connection to another gene in the IPKB. A score, that takes into account the number of Network Eligible Genes and the size of the networks, was calculated using a Fisher's exact test as the negative log of the probability that the genes within that network are associated by chance. Gene-enrichment of networks (network score), measured in IPA, using a modified Fisher's exact test was used to rank biological significance of gene function networks in relation to PTD.

In exploratory analysis, we applied Partek's single-level cross-validation method (Partek^® ^software, Partek Inc., St. Louis, MO) using a portion of the samples in each group as training dataset to generate a candidate signature gene list, and tested them on the rest of the arrays as the testing dataset to evaluate if the gene list would correctly identify array's group.

Common regulatory sequences for the differentially expressed genes as well as their cognate regulators (transcription factors (TFs)) were searched using ConTra (conserved transcription factor binding sites, TFBs) and MAPPER. ConTra is a promoter alignment analysis tool for identification of transcription factor binding sites across species. MAPPER (multi-genome analysis of positions and patterns of elements of regulation) is a platform for the computational identification of transcription factor binding sites (TFBSs) in multiple genomes which uses an innovative technique that combines TRANSFAC and JASPAR data with the search power of profile hidden Markov models. Since genes with similar expression profiles are likely to encode interacting proteins, we also evaluated protein-protein interactions (PPI) using Cytoprophet, a Cystoscape plug-in for protein and domain interaction network inference and results of our gene expression study (protein counterparts of differentially expressed genes) [33].

In the confirmatory RT-qPCR study, we repeated fold change analysis to evaluate differential expression measured using RT-qPCR for the selected genes from our set of differentially expressed genes in the microarray experiment. In this analysis, gene expression normalization (initially using RHOA gene) was done by determining delta Ct, where ΔCt = average Ct_target - average Ct_normalizer. Then the difference of mean Ct values between test and control was determined, ΔΔCt = ΔCt_control - ΔCt_test. Determination of fold Change (FC) was based on FC = Primer Efficiency^ΔΔCt^. True fold change was represented by the following: if FC > 1, true fold change = FC; if FC< 1, true fold change = -1/FC.

## Results

In this study, we investigated 14 PTD cases and 16 controls (Table 1). Blood was collected at 16 weeks of gestation, on average, comparably between cases and controls. A third of PTD cases followed spontaneous onset of labor while a third each followed preterm rupture of membrane and medically indicated labor induction and/or Cesarean delivery. Most study participants were white. Higher rates of preeclampsia, and chronic hypertension as well as smoking during the current pregnancy were observed for PTD cases compared with controls. PTD cases had also higher pre-pregnancy body mass index (BMI) compared with term controls.

**Table 1:** Characteristics of study population

Characteristics	Preterm cases (N = 14)	Term controls (N = 16)
GA at delivery, weeks*	32.3 (2.1)	39.1 (0.8)
GA at blood collection, weeks*	16.5 (2.1)	15.8 (1.4)
		
**Preterm delivery types**		
**Spontaneous labor**	4 (28.6)	0 (0.0)
**Preterm PROM**	5 (35.7)	0 (0.0)
**Other medically indicated**	5 (35.7)	0 (0.0)
		
Maternal Age, years*	33.0 (4.2)	33.1 (3.8)
**20-34 years**	8 (57.1)	9 (56.3)
**35 and above years**	6 (42.9)	7 (43.7)
		
**Race**		
**White**	8 (57.1)	15 (93.7)
**African American**	2 (14.3)	0 (0.0)
**Other**	4 (28.6)	1 (6.3)
		
**Pre-gestational BMI, kg/m**^2*^	29.6 (11.9)	23.8 (6.2)
		
**Multiparous**	6 (42.9)	9 (56.3)
**Multigravida**	10 (71.4)	12 (75.0)
**Cesarean delivery**	4 (28.6)	2 (12.5)
**Smoked in pregnancy**	2 (14.3)	1 (6.3)
**Preeclampsia**	4 (28.6)	0 (0.0)
**Gestational diabetes**	2 (14.3)	2 (12.5)
**Diabetes mellitus**	0 (0.0)	0 (0.0)
**Chronic hypertension**	4 (28.6)	1 (6.3)
**Chorioamnionitis**	1 (7.1)	2 (12.5)
**Abruptio placenta**	1 (7.1)	0 (0.0)
**IUGR**	0 (0.0)	0 (0.0)
**Cervical insufficiency**	1 (7.1)	0 (0.0)

*Mean, otherwise n (%)

Abbreviations: GA: gestational age, PROM: premature rupture of membrane, IUGR: intrauterine growth retardation, BMI: body mass index; kg/m^2^: kilogram/meter^2^

**Figure 1:**
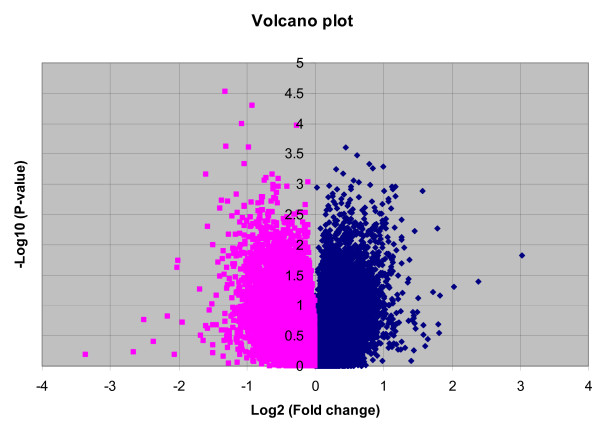
**Volcano plot of gene expression differences between cases and controls**. Volcano plot distributions of fold change (log2 [fold change]) (X-axis) and Student's t-test p-values (-log10 [p-value]) (Y-axis)

A volcano plot that demonstrates the Student's t-test based p-value and fold change differences in gene expression between the 14 PTD cases and 16 controls is shown in Figure 1. There are a total of ~47,400 probe sets (transcripts) representing >38,500 genes on the GeneChip. Of these, 1579 probe sets representing 209 genes were up or down regulated (99 up regulated and 110 down regulated) in PTD cases using the criteria of p-value < 0.05 and absolute fold change > 1.5 (Additional File 1). The resulting gene list included strong candidate genes such as LOC442421, a gene similar to prostaglandin E receptor 4, prolactin, Protein C, cystathionine-beta-synthase, tumor necrosis factor receptor superfamily, member 13B, and FLT1 as well as a number of novel genes with potential significance such as TEX1 (testis expressed gene 1), Transgelin and methylthioadenosine phosphorylase.

**Figure 2:**
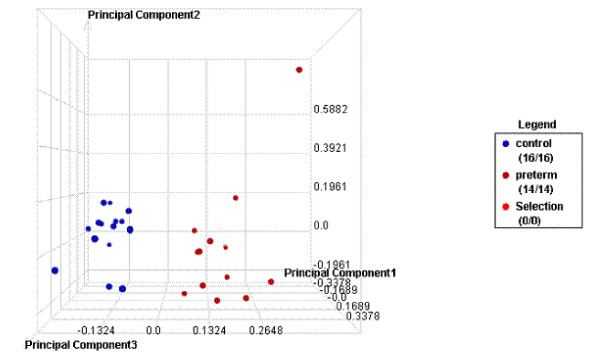
**Principal component analyses**. Principal component analysis results of all samples (14 cases and 16 controls) using 209 differentially expressed genes. (Red/right: cases, Blue/left: controls).

**Figure 3:**
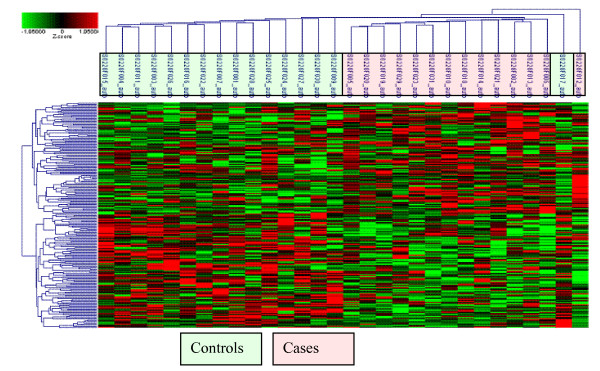
**Heat map illustration of samples and selected differentially expressed genes**. Selected genes (N = 209) differentially expressed (upregulated: shades of red and downregulated: shades of green) in whole blood associated with preterm delivery (rows) and participants (columns, cases = pink and controls = green) grouped according to level and nature of gene expression (genes) and similarity of expression profiles (participants) and subjected to hierarchical tree clustering.

PCA using these 209 genes and the 30 arrays was able to clearly separate the two groups of PTD cases and term delivery controls (Figure 2). Similarly, in the 2-D clustering analysis, as shown in the heat map in Figure 3, the 209 differentially expressed genes grouped most of cases and controls separately. In the exploratory analysis using Partek's automated model selection method, overall, 1-level cross validation rates based on over 10 testing sets were between 65-69%. In other words, a gene list generated from each training dataset gave a correct prediction rate of around 65-69% (about two out of three).

Results from functions and functional relationships of the differentially expressed genes using IPA are shown in Table 2 and Additional Files 2, 3, 4, 5, 6 and 7. Genes in our list of differentially expressed genes over represented six networks. The top network (Score = 49) as well as another network among significantly represented networks (Score = 19) has functions in the immune system and inflammation, lymphatic system, tissue morphology and hematological system development and function. Other networks that were also well represented included those related to development (cellular and organ development involving the nervous system, reproductive system and cardiovascular system) and metabolism (lipid, carbohydrate and amino acid). In addition, networks in the IPA analysis provide supportive evidence for genes such as NFKB, P38MAPK, Mapk, Akt, Vegf, retinoic acid, HOXA9, TNF, dihydrotestosterone, thyroid hormone, CDKNA1, sulfotransferase, Ggt, Oleic acid, progesterone, CCL2 and KNG which were not differentially expressed in our data set but could play significant roles in PTD.

**Table 2:** Gene networks* overrepresented by differentially expressed genes associated with preterm delivery

Genes in Network	Score	Focus genes	Functions
			

14-3-3, **AKAP12**, Akt, **ALK**, Ap1, **BIRC5**, CaMKII, **CD53, CD69, CDC25A, CYP2E1**, E2f, **FAM46A, FCER1A, FLT1, GATA2, HDC**, Histone h3, **HMGA2, IGHM**, Mapk, **NEFL**, NFkB, P38 MAPK, PDGF BB, **PRL, PROC, RGS7, SYNPO2, TAGLN, TIRAP, TNFRSF13B, TOP2A**, Vegf, **ZBTB10**	49	24	Immune and Lymphatic System Development and Function, Tissue Morphology, Hematological System Development and Function

BMI1, **CBS, CDCA5**, CDKN2A, DHX8, DHX15, EEF1A1, **EXOSC4**, GAS7, GFM1, GPRC5A, **HADHA, HOXA1, HOXA2**, HOXA9, HOXA3 (includes EG:3200), HOXB2, HOXB9, HOXC5, **ID4, KIF1B, MBNL1, MEG3 (includes EG:55384)**, MEIS2, MPHOSPH1, **P4HA2, PBX1**, PBXIP1, RCBTB2, **RECQL4**, retinoic acid, SKIV2L, **TLX2**, TMSB10, WSB1	24	14	Cellular Development, Nervous System Development and Function, Cell Cycle

AK2, androsterone, CCNI, CDK2, **CKB**, dihydrotestosterone, ECH1, **GPD2**, HIPK1, HSD17B6, KAP, **LOC645619**, MAK, ME1, **MMP17, MS4A3, NCKAP1**, NR6A1, RASAL2, SAMD4A, **SEC22B, SLC16A7**, SLC1A4, **SLC45A3, SMURF1, SRGAP2**, SVIL, TALDO1, thyroid hormone, TNF, **WDR90**, WT1, WTAP, YWHAG, **ZNF7**	22	13	Organ Development, Organ Morphology, Reproductive System Development and Function

**ABP1**, AKT1, amino acids, **ANK3, CDKN3**, CDKN1A, **CHST13**, EGF, HGF, HS3ST2, HS3ST5, HS3ST6, HS3ST3A1, HS3ST3B1, HS3ST4 (includes EG:9951), MYBL2, NEK4, **NUSAP1, PCDHGA11, PCDHGC3, PELO, PRKAA1, RAMP1**, SMP2A, **SPG20**, sulfotransferase, SULT1A2, SULT1A4, SULT1B1, SULT1C2, SULT1C4, SULT4A1, TERT, **UST, ZNF622**	22	13	Carbohydrate Metabolism, Amino Acid Metabolism, Post-Translational Modification

**ABCB9**, ACSBG1, ACSL3, ACSL4, ACSL5, **ACSL6**, ATP, butyric acid, C1QTNF2, **CPA3, DDAH1**, Ggt, GGT2, GGT3, GGT6, **GGTL3**, GGTL4, GGTLA1, GGTLA4, HOM-TES-103, IL13, JARID1A, KRAS, **MTAP**, NFYB, NR3C1, oleic acid, **PITRM1, RERE, RPL41**, **SLC26A6**, SLC7A5, ST8SIA4, **TRIM45, ZNF610**	19	12	Lipid Metabolism, Small Molecule Biochemistry, Molecular Transport

ACE2, AGT, BDKRB1, CCL2, **CD177**, CEBPB, CIITA, **CRYBB2**, CSF3, **DARC**, EMR1, ENPEP, EP300, HDAC1 (includes EG:3065), HLA-DOA, **HLA-DOB**, HLA-DQB1, HOPX, **HP**, IK, INMT, KNG1 (includes EG:3827), **LPP, MCOLN2**, MSH5, NAP1L1, PFKFB3, progesterone, **PTGDS**, RFXAP, **RRM2**, **SALL1**, SERBP1, **TMEM176A, XPNPEP1**	19	12	Cell-To-Cell Signaling and Interaction, Inflammatory Disease, Cardiovascular System Development and Function

*The networks were generated through the use of Ingenuity Pathways Analysis (Ingenuity^® ^Systems, http://www.ingenuity.com). Each gene identifier was mapped to its corresponding gene object in the Ingenuity Pathways Knowledge Base (IPKB). These genes were overlaid onto a global molecular network developed from information contained in the IPKB. Network enrichment is then assessed using a network score (negative log of p-values of Fisher tests). Focus genes (in bold) are genes identified in our list of differentially expressed genes. Networks shown here are those with network scores > 3.0.

**Figure 4:**
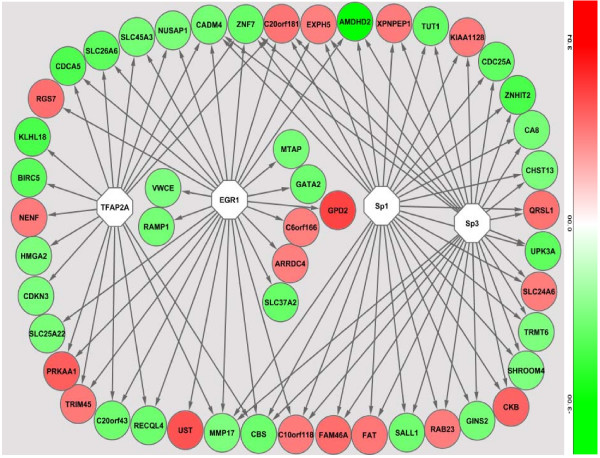
**Promoter analysis results of differentially expressed genes**. Inferred network of differentially expressed genes (Red = up regulated and Green = down regulated) in preterm delivery and transcription factors (White). Transcription factors were identified by their binding to over expressed promoter sequences in the differentially expressed genes.

**Figure 5:**
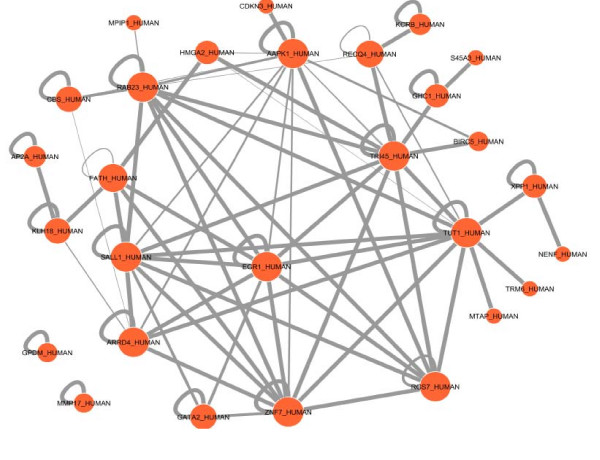
**Protein domain interactions of differentially expressed genes**. Predicted domain interactions of differentially expressed genes: For instance, EGR1 can potentially interact (not only with its targets gene motifs) with the domain of many of the genes forming the inferred network genes. The thickness of the edge shows extent of interaction.

In the promoter analysis, a number of common regulatory sequences, motifs, were identified. These sequences corresponded to binding sites of transcription factors TFAP2A (transcription factor AP2A), EGR1 (early growth response factor 1), Sp1 (specificity protein 1) and Sp3 (specificity protein 3) on genes that were differentially expressed in PTD cases compared to controls (Figure 4). In the predicted protein domain interaction analysis based on our gene expression results, evidence for significant interactions of the EGR1 protein domain with other putative protein domains is presented (Figure 5). Further, several protein domains of potential importance in PTD were identified such as TR145 and TUT1.

**Figure 6:**
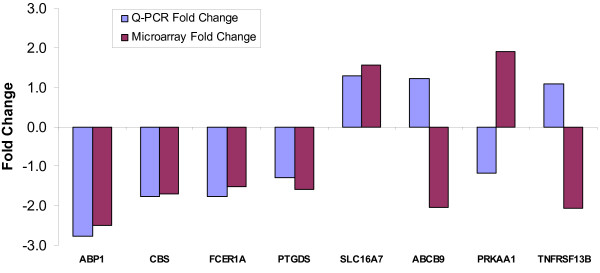
**Fold changes of selected genes in microarray and RT-QPCR experiments**.

In the confirmatory RT-qPCR experiment, expressions of nine genes (ABCB9, ABP1, CBS, FCER1A, PRKAA1, PTGDS, SLC16A7, TNFRSF13B and QRSL1) differentially expressed in PTD cases in the microarray experiment were evaluated. Three housekeeping genes (RHOA, KHDRBS1 and BAT1) were used in the experiment. For six of the nine genes, similar fold change differences and were observed between microarray and RT-qPCR expression measurements for an overall concordance rate of about 67% (Figure 6).

## Discussion

In this study, we show that early pregnancy peripheral blood gene expression is associated with PTD. Differentially expressed genes participate in diverse cellular pathways including immune function, inflammation, organ development, hematological system, metabolism and cell signaling underscoring the different pathophysiologic processes leading to PTD. Further, binding sites for transcription factors, EGR1, TFAP2A, Sp1 and Sp3 were overrepresented in promoter sequences of differentially expressed genes, potentially accounting for observed co-expressions.

Several pathophysiological mechanisms contribute to the occurrence of PTD following spontaneous preterm labor, PPROM or other medically indicated PTD. These include inflammation and infection, hormonal deregulation, decidual hemorrhage, uterine over-distension as well as local cervical abnormalities [34-39]. Previous uterine and cervical gene expression studies, mostly experimental, have shown that labor is associated with up/down regulation of genes involved in chemokine/cytokine activation, signal transduction, acute phase response, growth-differentiation, adhesion, extracellular matrix remodeling, contraction (calcium signaling, ion channels), complement and coagulation cascades, kinases, VEGF-family signaling, arginine and proline metabolism and apoptosis [35-39]. In the current study, supportive evidence for differential expression of genes participating in similar pathways in peripheral blood (e.g. FLT1) in early pregnancy is presented.

Proinflammatory cytokines and immune related chemokines stimulate prostaglandin production and uterine contraction through mechanisms that involve cycloxygenase-2 [34,40,41]. They act on cervical smooth muscles enhancing cervical ripening, activate matrix metalloproteinasess expression; and, increase protease production leading to extracellular matrix degradation, related cervical changes and premature rupture of membrane [34,40,41]. Both term and preterm labor are associated with a reduction in the percentage of monocytes expressing MHC class II depicting a pattern referred to as "monocyte hyporesponsiveness" or "immune paresis" [42]. HLA-DOB that suppresses peptide loading of MHC class II molecules by inhibiting HLA-DM, was upregulated in our study. Several other genes participating in immune function and inflammation (such as LILRB5, TIRAP, CD69 and CD53) were up regulated in our study.

Hormonal dysregulation (related to progesterone, prostaglandins and prolactin) has been associated with PTD. Progesterone withdrawal or a progestin-resistant environment may precede preterm labor [40]. Prostaglandin subtypes (E and F), released by the membranes in response to stretch and actions of pro-inflammatory cytokines, act not only upon the myometrium and cervix, but may also exert paracrine/autocrine effects on cell viability and matrix protein integrity [41]. On the other hand, other prostaglandins (such as subtype D) have roles in pregnancy maintenance [43]. LOC442421, a gene coding for a protein similar to the prostaglandin E4 receptor was up regulated in the current study. Further, PTGDS, a gene coding prostaglandin D2 synthase was down regulated in our study. The progesterone responsiveness of prostaglandin receptors is in part regulated by transcription factors belonging to the class of homeobox genes [40]. HOXA1, a previously described breast cancer related gene [44] that belongs to this class, was down regulated in our study. The PRL gene coding for prolactin, a growth hormone associated with lactation and that has been suggested for use in PTD prediction from cervicovaginal washings [45], was down regulated in our study.

PTD is frequently associated with abruption and/or decidual hemorrhage. These events result in intense local thrombin generation, binding to protease activated receptor-1 to stimulate decidual protease production promoting rupture of membranes and myometrial contractions to induce PTD [41]. Genes participating in the coagulation cascade including PROC and VWCE were differentially expressed in peripheral blood in our study.

Cervical remodeling (including softening, ripening, dilation and labor) associated with PTD is related to collagen solubility [46-49]. Since, cervix is composed of extracellular matrix components such as collagen, elastin, proteoglycans and others, systemic effects can influence cervical remodeling [46,47]. Fortunato et al demonstrated an increased mRNA expression for MMP2, MMP9, and MT1-MMP and a decreased expression for TIMP2 in prematurely ruptured membranes [48]. Suggestions for further studies to determine steps in collagen synthesis, cross-linking and fibril assembly that may be altered in early pregnancy have been put forth [47]. Our study has identified a number of potential genes such as COL23A1, P4HA2, participating in collagen metabolism and up regulated in peripheral blood in early pregnancy. Myometrial activation leading to PTD may result from the coordinated expression of a cassette of contraction-associated proteins that include ion channels (calcium and potassium), agonist receptors (i.e. oxytocin and prostaglandin) and gap junctions [50,51]. A number of genes involved in ion channel function regulation (S100A5, SLC24A6, KCNMB4, KCNH2 and ABP1) were differentially expressed in our study.

Labor involves a number of interacting pathomechanisms that are integrated by various regulatory mechanisms involving upstream regulators and/or transcription factors [52]. These regulators include a) basic domains (e.g. AP-1, cAMP, CRE) b) zinc coordinating DNA binding domains (e.g. SP1 and nuclear factors such as PPAR) c) homeo-domain transcription factors and d) beta-scaffold transcription factors (e.g. NFAT, NFKB and STAT) [52]. For instance, NFKB regulates functional progesterone withdrawal, pro-labor mediators such as cytokines, phospholipid metabolites and release of ECM remodeling enzymes [52]. In our study, direct (e.g. GATA2) and indirect (in pathway analysis, e.g. NFKB) evidence for involvement of these regulatory transcription factors in PTD is presented. For instance strong evidence for role of EGR1, from both promoter analysis and protein-protein-domain interaction analysis, supporting previous reports [38], is presented. EGR1 can be a potential target for enhancing preventive, early prediction or therapeutic efforts in PTD.

Previous gene expression studies investigating preterm labor and delivery were conducted on cervical and uterine tissue, mostly post partum under experimental settings. While evidence for changes in gene expression preceding clinical signs of labor have been put forward [51], to our knowledge, we are not aware of any "published study" that investigated gene expression in peripheral blood in early pregnancy to predict PTD. The timing of activation of each cascade of activities related to PTD [37] is not clear. Our study provides hypothesis-generating evidence for PTD risk factors and their interactions, particularly for those involving systemic processes. While PTD remains a significant public health problem, methodological limitations have hindered research in this area. Development of methods utilizing use of peripheral blood for investigating risk factors of PTD has great potential in understanding pathophysiologic pathways and predictive applications [53]. Other strengths of our study include the following. First, the study was conducted nested within a larger well-characterized prospective pregnancy cohort. Second, we used appropriate measures to collect and stabilize maternal antepartum peripheral blood samples for isolating high quality pure mRNA. Third, we conducted extensive evaluation of differentially expressed genes using pathway, promoter and protein-protein-domain interaction analysis using powerful statistical and bioinformatics tools to gain insights into processes underlying the pathophysiology of PTD.

Several potential limitations of our pilot study deserve mention. First, single measurement of peripheral blood gene expression may not provide a full picture of the gene expression changes associated with PTD. Second, since PTD is associated with a set of diverse pathological conditions, some of the observed changes may be related to the disease conditions and not to PTD specifically. Third, we may not have enough power to assess all expression changes associated with PTD. In absence of previous pilots for gene expression variations in PTD, we were not able to assess the statistical power of our study. Fourth, while we have frequency matched cases and controls for maternal age, and gestational age at blood draw, residual confounding by these variables or uncontrolled confounding by others (e.g. race) may account for these gene expression differences. Fifth, inter-individual variability of gene expression in peripheral blood related to differences in cellular constituency of collected samples may account for differences. Finally, since we are evaluating early pregnancy changes, most of our findings in gene expression changes are likely to identify systemic disease processes that start early in pregnancy and are associated with PTD. Nevertheless, given the substantial burden of PTD and the concordance of our findings with other cross sectional studies, our preliminary findings, if confirmed, are likely to have strong clinical and public health relevance.

In the confirmatory RT-qPCR experiment, 3 of the 9 genes (ABCB9, PRKAA1 and TNFRSF1) we evaluated did not have concordant expression measures with the microarray experiment. We investigated potential reasons (primer efficiencies, splice variants, choice of calibrator genes for normalization) for this discordance. Including primer efficiencies in recalculation of fold change differences did not improve the discordance. Of the three genes, one, PRKAA, had an Affymetrix target region located in a non-overlapping region with the ABI assay primer set. However, no information could be found in the public sequence or published domain for evidence of PRKAA splice variants. Further in-depth sequence analysis is needed to investigate this lack of overlap which may account for the discordance. Choice of alternative housekeeping genes (KHDRBS1 and BAT1) did not improve the concordance. The concordance observed in our study (67%) is comparable to the concordance from other published microarray studies, given the small number of genes assessed (nine). For example, two previous studies reported confirmation of 70% to 75% of gene expression trends determined by cDNA microarray analysis using real-time RT-PCR [54,55].

In summary, we have shown that PTD is associated with maternal early pregnancy blood gene expression changes. Maternal early pregnancy peripheral blood gene expression patterns may be useful for better understanding of the pathophysiology of PTD and risk prediction. Furthermore, since evaluating gene expression of peripheral blood in early pregnancy is practical, knowledge and experience gained from this area of research may allow for similar applications in other pregnancy disorders.

## Conclusion

Early pregnancy peripheral blood gene expression is associated with PTD. Differentially expressed genes participate in diverse cellular pathways including immune function, inflammation, organ development, hematological system, metabolism and cell signaling underscoring the different pathophysiologic processes leading to PTD. Further, binding sites for transcription factors, EGR1, TFAP2A, Sp1 and Sp3 were overrepresented in promoter sequences of differentially expressed genes, potentially accounting for observed co-expressions. Maternal early pregnancy blood gene expression patterns may be useful for better understanding of PTD pathophysiology and PTD risk prediction.

## Competing interests

A patent application, related to study findings reported in this manuscript, "Preterm delivery diagnostic assay (Williams MA and Enquobahrie DA" (Application No: 61049709, Filed, May 1, 2008) is pending. All authors declare that they have no other competing interests.

## Authors' contributions

Each author participated actively and sufficiently in study design (MW, DE, TS and CQ), data acquisition (MW, CQ, KS and ZG), data analysis (DE, CQ, SM, KS and ZG), data interpretation (all) and manuscript preparation (all). All authors have read and approved the submission of the final manuscript.

## Pre-publication history

The pre-publication history for this paper can be accessed here:

http://www.biomedcentral.com/1471-2393/9/56/prepub

## Supplementary Material

Additional file 1 Selected differentially* expressed genes in early pregnancy blood among women who delivered preterm. Selected genes differentially expressed in whole blood among women destined to deliver preterm in order of fold change values (fold), P-value: Students' t-test p-value. Click here for file

Additional file 2 **Network 1 identified in Ingenuity Pathway Analysis**. The networks were generated through the use of Ingenuity Pathways Analysis (Ingenuity^® ^Systems, http://www.ingenuity.com). Each gene identifier was mapped to its corresponding gene object in the Ingenuity Pathways Knowledge Base (IPKB) and overlaid onto a global molecular network developed from information contained in the IPKB. Colored genes are genes in our set of differentially expressed genes. Click here for file

Additional file 3 **Network 2 identified in Ingenuity Pathway Analysis**. The networks were generated through the use of Ingenuity Pathways Analysis (Ingenuity^® ^Systems, http://www.ingenuity.com). Each gene identifier was mapped to its corresponding gene object in the Ingenuity Pathways Knowledge Base (IPKB) and overlaid onto a global molecular network developed from information contained in the IPKB. Colored genes are genes in our set of differentially expressed genes. Click here for file

Additional file 4 **Network 3 identified in Ingenuity Pathway Analysis**. The networks were generated through the use of Ingenuity Pathways Analysis (Ingenuity^® ^Systems, http://www.ingenuity.com). Each gene identifier was mapped to its corresponding gene object in the Ingenuity Pathways Knowledge Base (IPKB) and overlaid onto a global molecular network developed from information contained in the IPKB. Colored genes are genes in our set of differentially expressed genes. Click here for file

Additional file 5 **Network 4 identified in Ingenuity Pathway Analysis**. The networks were generated through the use of Ingenuity Pathways Analysis (Ingenuity^® ^Systems, http://www.ingenuity.com). Each gene identifier was mapped to its corresponding gene object in the Ingenuity Pathways Knowledge Base (IPKB) and overlaid onto a global molecular network developed from information contained in the IPKB. Colored genes are genes in our set of differentially expressed genes. Click here for file

Additional file 6 **Network 5 identified in Ingenuity Pathway Analysis**. The networks were generated through the use of Ingenuity Pathways Analysis (Ingenuity^® ^Systems, http://www.ingenuity.com). Each gene identifier was mapped to its corresponding gene object in the Ingenuity Pathways Knowledge Base (IPKB) and overlaid onto a global molecular network developed from information contained in the IPKB. Colored genes are genes in our set of differentially expressed genes. Click here for file

Additional file 7 **Network 6 identified in Ingenuity Pathway Analysis**. The networks were generated through the use of Ingenuity Pathways Analysis (Ingenuity^® ^Systems, http://www.ingenuity.com). Each gene identifier was mapped to its corresponding gene object in the Ingenuity Pathways Knowledge Base (IPKB) and overlaid onto a global molecular network developed from information contained in the IPKB. Colored genes are genes in our set of differentially expressed genes. Click here for file
