# Supplementary material for: Early pregnancy peripheral blood gene expression and risk of preterm delivery: a nested case control study
Source: BMC Pregnancy Childbirth. 2009 Dec 10;9:56. doi: 10.1186/1471-2393-9-56 (PMC2799378; doi:10.1186/1471-2393-9-56)
Supplement: Additional file 1 — Selected differentially* expressed genes in early pregnancy blood among women who delivered preterm. Selected genes differentially expressed in whole blood among women destined to deliver preterm in order of fold change values (fold), P-value: Students' t-test p-value. [file 1471-2393-9-56-S1.DOC]

**Additional File 1 Selected differentially* expressed genes in early pregnancy blood among women who delivered preterm**

| **Accession Number** | **Gene symbol** | **Gene name** | **Fold *** | **P-value*** |
| --- | --- | --- | --- | --- |
| BF197664 | LOC442421 | similar to prostaglandin E receptor 4, subtype EP4; PGE receptor, EP4 subtype; prostaglandin E2 receptor | 8.18 | 0.0151 |
| AL049918 | DHRSX | Dehydrogenase/reductase (SDR family) X-linked | 5.24 | 0.0407 |
| AK000451 | FLJ20444 | hypothetical protein FLJ20444 | 4.08 | 0.0494 |
| NM_000496 | CRYBB2 | Crystallin, beta B2 | 2.97 | 0.0013 |
| U79250 | GPD2 | Glycerol-3-phosphate dehydrogenase 2 (mitochondrial) | 2.23 | 0.0051 |
| NM_000496 | CRYBB2 | Crystallin, beta B2 | 2.20 | 0.0267 |
| BC037847 | THADA | Thyroid adenoma associated | 2.19 | 0.0176 |
| NM_006840 | LILRB5 | Leukocyte immunoglobulin-like receptor, subfamily B (with TM and ITIM domains), member 2 | 2.16 | 0.0234 |
| NM_017518 | UIP1 | 26S proteasome-associated UCH interacting protein 1 | 2.10 | 0.0018 |
| BF439675 | CD69 | CD69 molecule | 2.10 | 0.0088 |
| AW418882 | UST | Uronyl-2-sulfotransferase | 2.05 | 0.0045 |
| AW134984 | LRRC8B | Leucine rich repeat containing 8 family, member B | 2.04 | 0.0038 |
| AI003930 | PITRM1 | Pitrilysin metallopeptidase 1 | 2.01 | 0.0048 |
| BC020226 | HLA-DOB | Major histocompatibility complex, class II, DO beta | 1.97 | 0.0181 |
| AI093572 | 214238_at | Clone DT1P1B6 mRNA, CAG repeat region | 1.95 | 0.0289 |
| BG619261 | PELO | Pelota homolog (Drosophila) | 1.91 | 0.0217 |
| BC012622 | PRKAA1 | Protein kinase, AMP-activated, alpha 1 catalytic subunit | 1.90 | 0.0344 |
| AI652000 | KCNH2 | Potassium voltage-gated channel, subfamily H (eag-related), member 2 | 1.88 | 0.0188 |
| AI690433 | COL23A1 | Collagen, type XXIII, alpha 1 | 1.87 | 0.0165 |
| BG150083 | TIRAP | Toll-interleukin 1 receptor (TIR) domain containing adaptor protein | 1.85 | 0.0258 |
| NM_001823 | CKB | Creatine kinase, brain | 1.83 | 0.0368 |
| AI022073 | CD53 | CD53 molecule | 1.82 | 0.0078 |
| NM_002962 | S100A5 | S100 calcium binding protein A5 | 1.81 | 0.0455 |
| T90358 | ZNF540 | yd43b08.s1 Soares fetal liver spleen 1NFLS Homo sapiens cDNA clone IMAGE:110967 3', mRNA sequence. | 1.80 | 0.0344 |
| AW450626 | C1orf131 | Chromosome 1 open reading frame 131 | 1.79 | 0.0069 |
| BE221330 | LOC389833 | similar to hypothetical protein MGC27019 | 1.78 | 0.0198 |
| NM_002588 | PCDHGC3 | Protocadherin gamma subfamily C, 3 | 1.77 | 0.0019 |
| W72333 | FLJ21657 | hypothetical protein FLJ21657 | 1.76 | 0.0055 |
| AF075104 | ZNF622 | Zinc finger protein 622 | 1.76 | 0.0321 |
| AL050106 | NHEJ1 | Nonhomologous end-joining factor 1 | 1.76 | 0.0058 |
| AV729634 | DNAJC6 | DnaJ (Hsp40) homolog, subfamily C, member 6 | 1.75 | 0.0273 |
| AL139377 | SPG20 | Human DNA sequence from clone RP11-251J8 on chromosome 13 Contains 2 novel genes, the KIAA0610 gene and a CpG island, complete sequence. | 1.74 | 0.0065 |
| AC006033 | STARD3NL | Homo sapiens BAC clone RP11-121A8 from 7, complete sequence. | 1.74 | 0.0010 |
| AA526820 | FAM46A | Family with sequence similarity 46, member A | 1.73 | 0.0480 |
| AW024499 | LOC339929 | hypothetical protein LOC339929 | 1.72 | 0.0059 |
| NM_002924 | RGS7 | Regulator of G-protein signalling 7 | 1.70 | 0.0203 |
| R49295 | PCDH9 | Protocadherin 9 | 1.68 | 0.0368 |
| AW629527 | FAM79B | Family with sequence similarity 79, member B | 1.68 | 0.0432 |
| BC006356 | TLX2 | T-cell leukemia homeobox 2 | 1.67 | 0.0138 |
| R67695 | FMN1 | Formin 1 | 1.67 | 0.0044 |
| AL136679 | QRSL1 | Glutaminyl-tRNA synthase (glutamine-hydrolyzing)-like 1 | 1.66 | 0.0024 |
| AK026281 | TRPM6 | Transient receptor potential cation channel, subfamily M, member 6 | 1.65 | 0.0321 |
| AI379784 | FLJ33630 | hypothetical protein LOC644873 | 1.65 | 0.0417 |
| U63828 | C20orf181 | Chromosome 20 open reading frame 181 | 1.65 | 0.0037 |
| AV727634 | ACSL6 | AV727634 HTC Homo sapiens cDNA clone HTCAYH08 5', mRNA sequence. | 1.64 | 0.0478 |
| NM_007167 | ZMYM6 | Zinc finger, MYM-type 6 | 1.64 | 0.0155 |
| AA732240 | MBNL1 | Muscleblind-like (Drosophila) | 1.63 | 0.0090 |
| AW300612 | 243947_s_at | Transcribed locus | 1.63 | 0.0100 |
| NM_018470 | C10orf110 | Chromosome 10 open reading frame 110 | 1.63 | 0.0071 |
| AA843962 | TMEM16E | Transmembrane protein 16E | 1.63 | 0.0436 |
| AI692560 | C22orf35 | Hypothetical protein LOC150271 | 1.62 | 0.0202 |
| AA776810 | ZNF610 | Zinc finger protein 610 | 1.62 | 0.0101 |
| NM_004199 | P4HA2 | Procollagen-proline, 2-oxoglutarate 4-dioxygenase (proline 4-hydroxylase), alpha polypeptide II | 1.62 | 0.0027 |
| NM_005245 | FAT | FAT tumor suppressor homolog 1 (Drosophila) | 1.61 | 0.0439 |
| AI290561 | C21orf2 | Transcribed locus | 1.61 | 0.0162 |
| AJ012680 | C1orf5 | Homo sapiens gene encoding hypothetical protein with HTH motif. | 1.61 | 0.0053 |
| N93197 | FLJ44606 | hypothetical gene supported by AK126569 | 1.61 | 0.0242 |
| AA872471 | SNRPA1 | Small nuclear ribonucleoprotein polypeptide A' | 1.60 | 0.0049 |
| BF508344 | FLJ12993 | hypothetical LOC441027 | 1.60 | 0.0175 |
| AA149648 | FLT1 | Fms-related tyrosine kinase 1 (vascular endothelial growth factor/vascular permeability factor receptor) | 1.59 | 0.0085 |
| BG236136 | LOC730249 | Transcribed locus, strongly similar to XP_292184.4 PREDICTED: similar to immune-responsive gene 1 [Homo sapiens] | 1.59 | 0.0415 |
| AL833376 | FLJ43944 | FLJ43944 protein | 1.58 | 0.0112 |
| AI793200 | TRIM45 | Tripartite motif-containing 45 | 1.58 | 0.0379 |
| AW339812 | N/A | CDNA FLJ39461 fis, clone PROST2011660 | 1.58 | 0.0283 |
| AW629478 | RERE | Arginine-glutamic acid dipeptide (RE) repeats | 1.58 | 0.0331 |
| AL356115 | KIAA1128 | Human DNA sequence from clone RP11-486O22 on chromosome 10 Contains the 3' end of a novel gene (KIAA1128, FLJ14262, FLJ25809) , the 5' end of a Siah-interacting protein (SIP) (calcyclin binding protein) pseudogene, ribosomal protein S3A pseudogene 5 (RPS3AP5) and two CpG islands, complete sequence. | 1.57 | 0.0344 |
| AI658662 | SYNPO2 | Synaptopodin 2 | 1.57 | 0.0264 |
| AI359676 | SH3GL3 | Transcribed locus | 1.57 | 0.0230 |
| AW609310 | XPNPEP1 | X-prolyl aminopeptidase (aminopeptidase P) 1, soluble | 1.57 | 0.0130 |
| X64978 | OR2L2 | Olfactory receptor, family 2, subfamily L, member 2 | 1.57 | 0.0259 |
| AW954477 | SUCLG1 | Succinate-CoA ligase, GDP-forming, alpha subunit | 1.56 | 0.0066 |
| AI984136 | NENF | Neuron derived neurotrophic factor | 1.56 | 0.0248 |
| AI800806 | 1560373_a_at | CDNA FLJ34680 fis, clone LIVER2003524 | 1.56 | 0.0270 |
| AW975728 | SLC16A7 | Solute carrier family 16 (monocarboxylic acid transporters), member 7 | 1.56 | 0.0125 |
| AI732542 | ZBTB10 | ni36g03.x5 NCI_CGAP_Lu1 Homo sapiens cDNA clone IMAGE:978964 3' similar to contains Alu repetitive element;contains element TAR1 TAR1 repetitive element ; mRNA sequence. | 1.56 | 0.0009 |
| BC034999 | KATNAL2 | Katanin p60 subunit A-like 2 | 1.56 | 0.0166 |
| AK024739 | C10orf118 | Chromosome 10 open reading frame 118 | 1.55 | 0.0016 |
| AI263819 | LOC653464 | similar to SLIT-ROBO Rho GTPase-activating protein 2 (srGAP2) (Formin-binding protein 2) | 1.55 | 0.0493 |
| BC012928 | C6orf150 | Chromosome 6 open reading frame 150 | 1.54 | 0.0264 |
| AY083533 | MCOLN2 | Mucolipin 2 | 1.54 | 0.0369 |
| AF161486 | RAB23 | RAB23, member RAS oncogene family | 1.54 | 0.0444 |
| AL390180 | TNRC17 | MRNA; cDNA DKFZp761L149 (from clone DKFZp761L149) | 1.54 | 0.0048 |
| AA280904 | C9orf39 | Chromosome 9 open reading frame 39 | 1.54 | 0.0298 |
| AI339568 | SLC24A6 | Solute carrier family 24 (sodium/potassium/calcium exchanger), member 6 | 1.54 | 0.0062 |
| AL537457 | NEFL | Neurofilament, light polypeptide 68kDa | 1.53 | 0.0409 |
| AK025047 | FLJ21394 | Homo sapiens cDNA: FLJ21394 fis, clone COL03536. | 1.53 | 0.0452 |
| AI927971 | MGC13034 | hypothetical protein MGC13034 | 1.53 | 0.0003 |
| AB014524 | EXPH5 | Exophilin 5 | 1.53 | 0.0373 |
| AF036973 | C6orf12 | Chromosome 6 open reading frame 12 | 1.52 | 0.0217 |
| AV701177 | ARRDC4 | Arrestin domain containing 4 | 1.52 | 0.0237 |
| NM_020960 | GPR107 | G protein-coupled receptor 107 | 1.52 | 0.0208 |
| BM918074 | LOC388796 | hypothetical LOC388796 | 1.52 | 0.0235 |
| AI684551 | AUTS2 | Autism susceptibility candidate 2 | 1.51 | 0.0068 |
| NM_013436 | NCKAP1 | NCK-associated protein 1 | 1.51 | 0.0157 |
| AW001754 | NEGR1 | Neuronal growth regulator 1 | 1.51 | 0.0201 |
| AL832163 | LOC441376 | AARD protein | 1.51 | 0.0060 |
| AI742378 | C6orf166 | Chromosome 6 open reading frame 166 | 1.51 | 0.0138 |
| BC015906 | FMN1 | Homo sapiens formin 1, mRNA (cDNA clone IMAGE:3922558), **** WARNING: chimeric clone ****. | 1.51 | 0.0491 |
| AI247368 | 242798_at | Transcribed locus | 1.50 | 0.0457 |
| AI862096 | VWCE | Von Willebrand factor C and EGF domains | -1.50 | 0.0172 |
| AW501360 | FAM22B | Family with sequence similarity 22, member B | -1.50 | 0.0356 |
| AB033028 | SHROOM4 | KIAA1202 protein | -1.51 | 0.0158 |
| BC005912 | FCER1A | Fc fragment of IgE, high affinity I, receptor for; alpha polypeptide | -1.51 | 0.0017 |
| NM_003483 | HMGA2 | High mobility group AT-hook 2 | -1.51 | 0.0115 |
| CA448125 | 1557051_s_at | Homo sapiens, clone IMAGE:5019307, mRNA | -1.52 | 0.0182 |
| BE897074 | CGI-09 | CGI-09 protein | -1.52 | 0.0317 |
| S79910 | HOXA1 | Homeobox A1 | -1.52 | 0.0043 |
| AA670271 | N/A | af25e10.s1 Soares_total_fetus_Nb2HF8_9w Homo sapiens cDNA clone IMAGE:1032714 3', mRNA sequence. | -1.52 | 0.0154 |
| AA830326 | LOC644173 | hypothetical protein LOC644173 | -1.52 | 0.0338 |
| BE674989 | C1orf75 | Chromosome 1 open reading frame 75 | -1.52 | 0.0134 |
| AI701857 | 243359_at | Transcribed locus | -1.52 | 0.0316 |
| AV721013 | C19orf36 | Chromosome 19 open reading frame 36 | -1.52 | 0.0012 |
| NM_006735 | HOXA2 | Homeobox A2 | -1.52 | 0.0010 |
| AA677272 | CHST13 | Carbohydrate (chondroitin 4) sulfotransferase 13 | -1.52 | 0.0077 |
| AW157094 | ID4 | Inhibitor of DNA binding 4, dominant negative helix-loop-helix protein | -1.53 | 0.0483 |
| NM_182628 | CCDC37 | gb:NM_017674.1 /DB_XREF=gi:8923119 /GEN=FLJ20123 /FEA=FLmRNA /CNT=3 /TID=Hs.272232.0 /TIER=FL /STK=0 /UG=Hs.272232 /LL=54824 /DEF=Homo sapiens hypothetical protein FLJ20123 (FLJ20123), mRNA. /PROD=hypothetical protein FLJ20123 /FL=gb:NM_017674.1 | -1.53 | 0.0220 |
| NM_005143 | HP | Haptoglobin | -1.53 | 0.0150 |
| NM_016155 | MMP17 | Matrix metallopeptidase 17 (membrane-inserted) | -1.53 | 0.0399 |
| AU146834 | PBX1 | AU146834 HEMBB1 Homo sapiens cDNA clone HEMBB1001635 3', mRNA sequence. | -1.53 | 0.0481 |
| AF213040 | CDKN3 | Cyclin-dependent kinase inhibitor 3 (CDK2-associated dual specificity phosphatase) | -1.54 | 0.0341 |
| R55769 | N/A | yg89e01.s1 Soares infant brain 1NIB Homo sapiens cDNA clone IMAGE:40625 3', mRNA sequence. | -1.54 | 0.0231 |
| AF199364 | SMURF1 | SMAD specific E3 ubiquitin protein ligase 1 | -1.54 | 0.0007 |
| AI277101 | WDR90 | Hypothetical protein KIAA1924 | -1.55 | 0.0452 |
| NM_024698 | SLC25A22 | Solute carrier family 25 (mitochondrial carrier: glutamate), member 22 | -1.55 | 0.0217 |
| NM_018454 | NUSAP1 | Nucleolar and spindle associated protein 1 | -1.55 | 0.0181 |
| NM_000948 | PRL | Prolactin | -1.55 | 0.0469 |
| AL050350 | DDO | Human DNA sequence from clone RP1-261K5 on chromosome 6q21-22.1 Contains the 3' end of the SLC22A16 gene for solute carrier family 22 (organic cation transporter) member 16, the DDO gene for D-aspartate oxidase, the 5' part of a novel gene and two CpG islands, complete sequence. | -1.55 | 0.0395 |
| AK025919 | HADHA | Hydroxyacyl-Coenzyme A dehydrogenase/3-ketoacyl-Coenzyme A thiolase/enoyl-Coenzyme A hydratase (trifunctional protein), alpha subunit | -1.55 | 0.0290 |
| NM_080819 | GPR78 | G protein-coupled receptor 78 | -1.56 | 0.0019 |
| AI860021 | N/A | wm22h08.x1 NCI_CGAP_Ut4 Homo sapiens cDNA clone IMAGE:2436735 3' similar to contains Alu repetitive element;contains element MER40 repetitive element ; mRNA sequence. | -1.56 | 0.0052 |
| L35848 | MS4A3 | Membrane-spanning 4-domains, subfamily A, member 3 (hematopoietic cell-specific) | -1.57 | 0.0416 |
| NM_030970 | MGC3771 | hypothetical protein MGC3771 | -1.57 | 0.0181 |
| AI423493 | GGTL3 | Gamma-glutamyltransferase-like 3 | -1.58 | 0.0365 |
| AU154125 | SEC22B | SEC22 vesicle trafficking protein homolog B (S. cerevisiae) | -1.59 | 0.0113 |
| AI217472 | PHF7 | PHD finger protein 7 | -1.59 | 0.0287 |
| NM_004056 | CA8 | Carbonic anhydrase VIII | -1.59 | 0.0030 |
| AK024712 | CSS3 | chondroitin sulfate synthase 3 | -1.59 | 0.0183 |
| AF109294 | MTAP | Methylthioadenosine phosphorylase | -1.59 | 0.0034 |
| NM_000954 | PTGDS | Prostaglandin D2 synthase 21kDa (brain) | -1.60 | 0.0127 |
| AK097628 | STMN3 | CDNA FLJ40309 fis, clone TESTI2029470 | -1.60 | 0.0029 |
| AF182276 | CYP2E1 | Cytochrome P450, family 2, subfamily E, polypeptide 1 | -1.60 | 0.0424 |
| BC003186 | GINS2 | GINS complex subunit 2 (Psf2 homolog) | -1.60 | 0.0216 |
| BC007257 | CBS | Cystathionine-beta-synthase | -1.60 | 0.0142 |
| U66559 | ALK | Anaplastic lymphoma kinase (Ki-1) | -1.60 | 0.0076 |
| BF114967 | AKAP12 | A kinase (PRKA) anchor protein (gravin) 12 | -1.60 | 0.0324 |
| BC001886 | RRM2 | Ribonucleotide reductase M2 polypeptide | -1.61 | 0.0338 |
| BE297946 | TMTC1 | Transmembrane and tetratricopeptide repeat containing 1 | -1.61 | 0.0182 |
| AK056624 | KCNH2 | Potassium voltage-gated channel, subfamily H (eag-related), member 2 | -1.61 | 0.0032 |
| AI082237 | TAGLN | Transgelin | -1.62 | 0.0040 |
| NM_020995 | HPR | Haptoglobin | -1.62 | 0.0206 |
| AA631143 | SLC45A3 | Solute carrier family 45, member 3 | -1.62 | 0.0155 |
| AK098249 | LOC283050 | hypothetical protein LOC283050 | -1.63 | 0.0496 |
| NM_005855 | RAMP1 | Receptor (calcitonin) activity modifying protein 1 | -1.63 | 0.0332 |
| NM_019858 | GPR162 | G protein-coupled receptor 162 | -1.63 | 0.0332 |
| AI653169 | AK3L1 | Transcribed locus | -1.64 | 0.0062 |
| AF152505 | PCDHGA11 | Protocadherin gamma subfamily C, 3 | -1.64 | 0.0425 |
| AL049748 | RBM9 | Human DNA sequence from clone RP1-41P2 on chromosome 22, complete sequence. | -1.64 | 0.0131 |
| AW204383 | IGSF4C | Immunoglobulin superfamily, member 4C | -1.64 | 0.0267 |
| AI820801 | LOC643982 | hypothetical protein LOC643982 | -1.66 | 0.0053 |
| BG171323 | ADD2 | Clone 23700 mRNA sequence | -1.66 | 0.0065 |
| NM_018022 | TMEM51 | Transmembrane protein 51 | -1.66 | 0.0368 |
| NM_002112 | HDC | Histidine decarboxylase | -1.67 | 0.0276 |
| NM_002968 | SALL1 | Sal-like 1 (Drosophila) | -1.67 | 0.0489 |
| NM_001149 | ANK3 | Ankyrin 3, node of Ranvier (ankyrin G) | -1.67 | 0.0060 |
| M87789 | IGHG3 | Immunoglobulin heavy constant gamma 1 (G1m marker) | -1.69 | 0.0409 |
| BC002769 | C20orf43 | Chromosome 20 open reading frame 43 | -1.70 | 0.0357 |
| BE613178 | CBS | Cystathionine-beta-synthase | -1.70 | 0.0353 |
| NM_004260 | RECQL4 | RecQ protein-like 4 | -1.71 | 0.0016 |
| AI571298 | EXOSC4 | Exosome component 4 | -1.71 | 0.0061 |
| AL561834 | TOP2A | Topoisomerase (DNA) II alpha 170kDa | -1.71 | 0.0116 |
| NM_001870 | CPA3 | Carboxypeptidase A3 (mast cell) | -1.71 | 0.0016 |
| AL563460 | GATA2 | GATA binding protein 2 | -1.72 | 0.0027 |
| AI672553 | AKAP12 | A kinase (PRKA) anchor protein (gravin) 12 | -1.73 | 0.0310 |
| BC028053 | LOC645677 | similar to ciliary rootlet coiled-coil, rootletin | -1.74 | 0.0081 |
| AA977975 | VMO1 | Vitelline membrane outer layer 1 homolog (chicken) | -1.76 | 0.0148 |
| NM_000312 | PROC | Protein C (inactivator of coagulation factors Va and VIIIa) | -1.76 | 0.0122 |
| AW779022 | TTLL4 | Tubulin tyrosine ligase-like family, member 4 | -1.76 | 0.0313 |
| AL137510 | KCNMB4 | Potassium large conductance calcium-activated channel, subfamily M, beta member 4 | -1.77 | 0.0045 |
| BF511276 | AKAP12 | A kinase (PRKA) anchor protein (gravin) 12 | -1.77 | 0.0178 |
| AI935644 | SLC37A2 | Solute carrier family 37 (glycerol-3-phosphate transporter), member 2 | -1.78 | 0.0116 |
| AK022466 | ZNF7 | Zinc finger protein 7 | -1.78 | 0.0389 |
| AI826833 | 241783_at | Transcribed locus | -1.78 | 0.0236 |
| BF433930 | DERL3 | Der1-like domain family, member 3 | -1.79 | 0.0277 |
| AL583533 | SLC7A10 | Transcribed locus | -1.80 | 0.0098 |
| AW196588 | LOXHD1 | Lipoxygenase homology domains 1 | -1.83 | 0.0209 |
| NM_022830 | RBM21 | RNA binding motif protein 21 | -1.84 | 0.0179 |
| AI343459 | CDC25A | Cell division cycle 25A | -1.84 | 0.0170 |
| BF055370 | MGC72075 | hypothetical protein MGC72075 | -1.85 | 0.0178 |
| AK092805 | LOC284757 | hypothetical protein LOC284757 | -1.86 | 0.0179 |
| NM_006953 | UPK3A | Uroplakin 3A | -1.87 | 0.0070 |
| AL078459 | DDAH1 | Human DNA sequence from clone RP4-621F18 on chromosome 1p11.4-21.3 Contains part of a novel gene, and the 3' end of the DDAH1 gene for dimethylarginine dimethylaminohydrolase 1, complete sequence. | -1.89 | 0.0203 |
| BF956762 | MEG3 | Maternally expressed 3 | -1.91 | 0.0202 |
| AF288410 | SLC26A6 | Solute carrier family 26, member 6 | -1.92 | 0.0252 |
| AF088044 | 1560978_at | Full length insert cDNA clone ZD58F01 | -1.94 | 0.0261 |
| AA648913 | BIRC5 | Baculoviral IAP repeat-containing 5 (survivin) | -1.94 | 0.0450 |
| NM_018487 | HCA112 | hepatocellular carcinoma-associated antigen 112 | -2.01 | 0.0269 |
| AI088361 | 239970_at | Transcribed locus | -2.04 | 0.0048 |
| BG427399 | KIF1B | Kinesin family member 1B | -2.05 | 0.0005 |
| NM_002036 | DARC | Duffy blood group, chemokine receptor | -2.05 | 0.0023 |
| BE504895 | ABCB9 | ATP-binding cassette, sub-family B (MDR/TAP), member 9 | -2.05 | 0.0069 |
| NM_012452 | TNFRSF13B | Tumor necrosis factor receptor superfamily, member 13B | -2.06 | 0.0024 |
| BE614410 | CDCA5 | Cell division cycle associated 5 | -2.06 | 0.0377 |
| NM_014205 | ZNHIT2 | Zinc finger, HIT type 2 | -2.13 | 0.0100 |
| BM141828 | KLHL18 | Kelch-like 18 (Drosophila) | -2.14 | 0.0065 |
| AB003476 | AKAP12 | A kinase (PRKA) anchor protein (gravin) 12 | -2.22 | 0.0015 |
| NM_001091 | ABP1 | Potassium voltage-gated channel, subfamily H (eag-related), member 2 | -2.50 | 0.0305 |
| NM_020406 | CD177 | gb:NM_020406.1 /DEF=Homo sapiens polycythemia rubra vera 1; cell surface receptor (PRV1), mRNA. /FEA=mRNA /GEN=PRV1 /PROD=polycythemia rubra vera 1; cell surfacereceptor /DB_XREF=gi:9966888 /UG=Hs.232165 polycythemia rubra vera 1; cell surface receptor /FL=gb:AF146747.1 gb:NM_020406.1 | -2.63 | 0.0025 |
| AY014285 | TEX101 | Testis expressed sequence 101 | -2.98 | 0.0050 |
| NM_015944 | AMDHD2 | Amidohydrolase domain containing 2 | -4.01 | 0.0183 |

*Selected genes differentially expressed in whole blood among women destined to deliver preterm in order of fold change values (fold), P-value: Students’ t-test p-value.

N/A=not available.
