# Supplementary material for: Early pregnancy peripheral blood gene expression and risk of preterm delivery: a nested case control study
Source: BMC Pregnancy Childbirth. 2009 Dec 10;9:56. doi: 10.1186/1471-2393-9-56 (PMC2799378; doi:10.1186/1471-2393-9-56)

# Figure 5 Network 5 identified in Ingenuity Pathway Analysis

Lipid metabolism, small molecule biochemistry, molecular transport

#
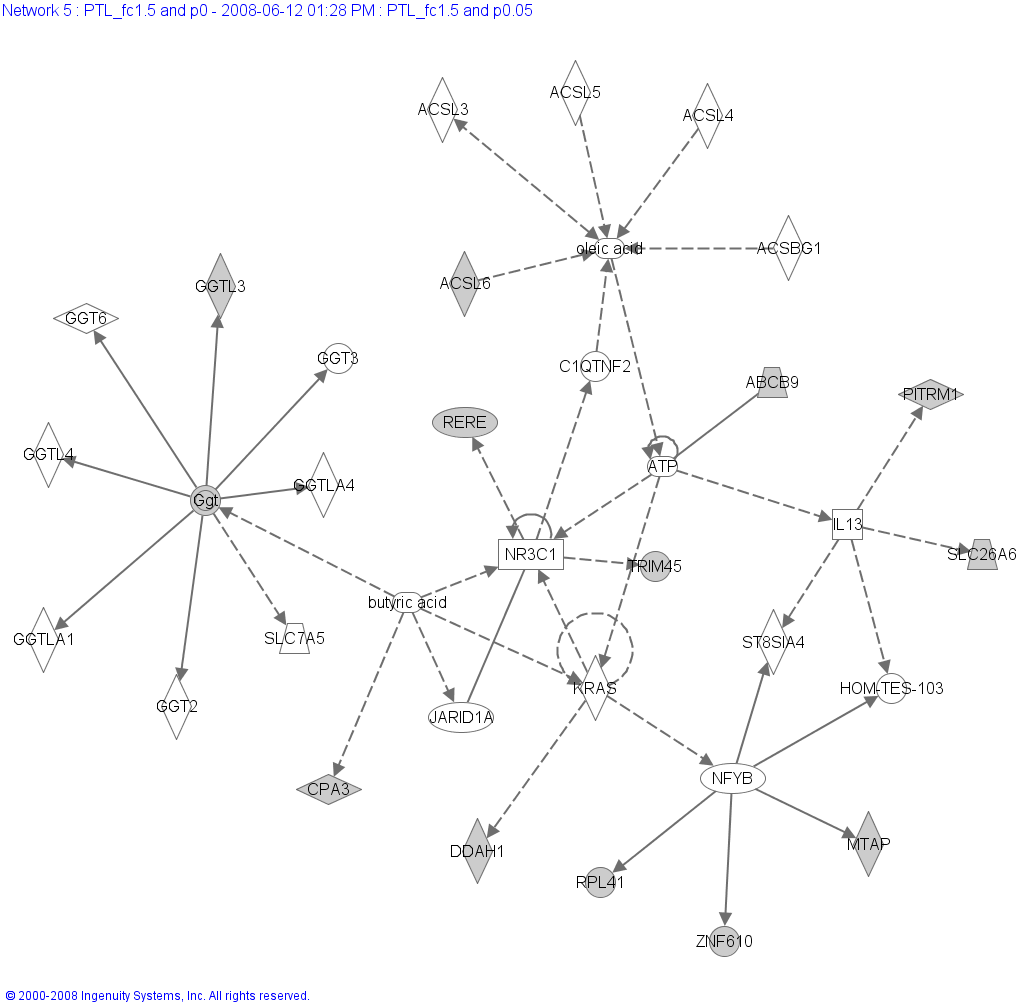

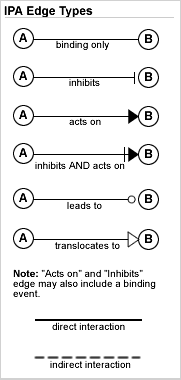

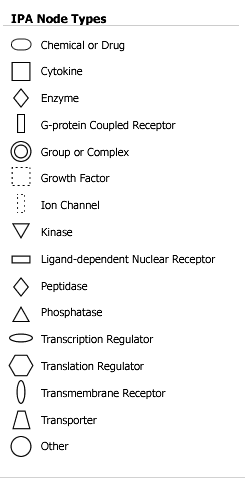

Supplement: Additional file 6 — Network 5 identified in Ingenuity Pathway Analysis. The networks were generated through the use of Ingenuity Pathways Analysis (Ingenuity® Systems, http://www.ingenuity.com). Each gene identifier was mapped to its corresponding gene object in the Ingenuity Pathways Knowledge Base (IPKB) and overlaid onto a global molecular network developed from information contained in the IPKB. Colored genes are genes in our set of differentially expressed genes. [file 1471-2393-9-56-S6.DOC]
